# Downregulation of Long Noncoding RNA LINC00261 Attenuates Myocardial Infarction through the miR-522-3p/Trinucleotide Repeat-Containing Gene 6a (TNRC6A) Axis

**DOI:** 10.1155/2021/6628194

**Published:** 2021-06-18

**Authors:** Chaoxin Jiang, Qing Zhao, Chenlong Wang, Minyan Peng, Guoqing Hao, Zhifeng Liu, Wenjin Fu, Kewei Zhao

**Affiliations:** ^1^Department of Clinical Laboratory, Guangdong Provincial Hospital of Integrated Traditional Chinese and Western Medicine, Foshan, Guangdong 528200, China; ^2^Department of Clinical Laboratory, The Third Affiliated Hospital, Guangzhou University of Chinese Medicine, Guangzhou, Guangdong 510240, China; ^3^Department of Laboratory Medicine, Nanhai Hospital, Southern Medical University, Foshan, Guangdong 528244, China; ^4^Department of Laboratory, Affiliated Houjie Hospital, Guangdong Medical College, Dongguan, Guangdong 523945, China

## Abstract

**Background:**

Myocardial infarction (MI) is cardiac tissue necrosis caused by acute and persistent ischemic hypoxia of the coronary arteries. This study is aimed at investigating the expression of long noncoding RNA (lncRNA) LINC00261 in MI and its effect on myocardial cells.

**Methods:**

qRT-PCR was performed to detect the expression levels of LINC00261, miR-522-3p, and TNRC6A in normal and MI cells. Western blotting analysis was performed to detect the expression of TNRC6A protein. Viability and apoptosis of myocardial cells after MI with the knockout of LINC00261 or TNRC6A were detected. The relationships among miR-522-3p, LINC00261, and TNRC6A in cardiomyocytes were evaluated using a double luciferase reporter gene assay. Hypoxic preconditioning in normal cells was used to construct a simulated MI environment to investigate the effect of LINC00261 on apoptosis of cardiac cells.

**Results:**

LINC00261 and TNRC6A were upregulated, while miR-522-3p was downregulated in coronary heart disease tissues with MI. Knockout of LINC00261 can increase the viability of cardiomyocytes and inhibit cell apoptosis. LINC00261 targets miR-522-3p in cardiomyocytes. In addition, miR-522-3p targets TNRC6A in cardiomyocytes. TNRC6A regulates cell viability and apoptosis of cardiomyocytes after MI, and TNRC6A-induced MI can be reversed by overexpression of miR-522-3p.

**Conclusions:**

LINC00261 downregulated miR-522-3p in cardiomyocytes after MI by directly targeting miR-522-3p. TNRC6A is the direct target of miR-522-3p. Our results indicated that LINC00261 might serve as a therapeutic target for the treatment of MI.

## 1. Introduction

Myocardial infarction (MI) is cardiac tissue necrosis caused by acute and persistent ischemic hypoxia of the coronary arteries [1]. It is one of the leading causes of death in the world, with a large population diagnosed as new MI cases every year [2–5]. The prognosis of acute MI is closely related to the size of the infarct size, complications, and treatment [6]. Most of the deaths occur within the first week, even worse, within 1 to 2 hours after symptoms begin [7–9]. Therefore, a considerable number of patients died of ventricular fibrillation before being hospitalized. In addition to serious arrhythmias, MI may also cause cardiogenic shock, heart failure, and heart rupture even after hospitalization [10, 11]. Therefore, finding new targets for the treatment of MI is of great importance.

Long noncoding RNAs (lncRNAs) are widely involved in biological processes such as cell metabolism, signal transduction, proliferation, differentiation, apoptosis, and cell death and are of great significance for the occurrence and development of cardiovascular diseases [12]. lncRNA is a type of RNA longer than 200 nt with no translation function [13]. With the rapid development of high-throughput sequencing technologies and microarray technology, various lncRNAs have been identified to exert important biological functions in heart diseases [14]. One study demonstrated that loss of function of LINC00261 results in impaired cardiovascular development [15]. However, the regulatory mechanisms of lncRNAs in MI are still not elusive.

Endogenous noncoding single-stranded microRNAs (miRNAs) are normally found in eukaryotes and can regulate multiple gene expression [16]. miRNA is about 18-25 nt long [17]. miRNAs can specifically recognize and bind to the 3′-untranslated region (3′-UTR) of the target gene, causing degradation of mRNAs or inhibition of translation of the targeted mRNAs, thereby exerting a negative regulatory effect and participating in cell proliferation, differentiation, immune regulation, and apoptosis [18]. A recent study showed that overexpression of LINC00261 could interact with miR-522-3p [19]. Our preliminary bioinformatics analysis results revealed that LINC00261 could bind with miR-522-3p. Trinucleotide repeat-containing gene 6a (TNRC6A) is an Argonaute-navigator protein for miRNA-mediated gene silencing in the nucleus [20]. Altered expression of TNRC6a and miR-21 in MI has been reported [21]. Our bioinformatics analysis results showed that miR-522-3p could interact with TNRC6A [19]. Therefore, we investigated the interactions among LINC00261, miR-522-3p, and TNRC6A in MI.

## 2. Methods

### 2.1. Mouse MI Model

A total of 100 male C57B/L6 mice aged 8-10 weeks old and weighed 18-25 g were purchased from the First Affiliated Hospital of Bengbu Medical College Animal Research Institute. The mice were anesthetized by intraperitoneal injection of avertin solution before surgery, fixed in supine position on mouse plate. The MI group included 80 mice, and the sham group included 20 mice. The ventilator was ligated to the anterior descending branch of the left coronary artery 3-4 mm as previously described [22]. Then, 3 d after the surgery, 5 mice died in the MI group and no mice died in the sham group. The myocardial tissues of 10 mice from the MI group and the sham group were harvested. In the following 4 weeks, 10 mice in the MI group died and the rest were housed for breeding for the following experiments. This study was approved by the Animal Ethics Committee of the Third Affiliated Hospital, Guangzhou University of Chinese Medicine. All procedures involving animals were conducted following the guidelines for the use of laboratory animals in China.

### 2.2. Cells and MI Induction

Cardiac cells H9c2 were provided by BeNa, China. Cells were incubated in DMEM with 100 U/ml penicillin, 0.1 mg/mL streptomycin, and 10% FBS at 37°C with 5% CO_2_. The MI-acute hypoxic-ischemic coronary disease *in vitro* model was established. The simulated MI environment of H9c2 cardiomyocytes was established by performing hypoxia pretreatment for 30 min. Interference experiment was performed after MI cardiac muscle cells were recovered with 95% O_2_% and 5% CO_2_ for 30 min.

### 2.3. Cell Transfection

Overexpression of the gene 6a (TNRC6A) containing a trinucleotide was achieved by transfecting pcDNA3.1-TNRC6A vector (Invitrogen, USA). miR-522-3p mimic, miR-522-3p inhibitor, and si-RNA of LINC00261 and TNRC6A were synthesized by GenePharma (Shanghai, China). Commercialized oligonucleotide siRNA (Shanghai, China) was used as the negative control (NC). Transfecting experiments were performed using Lipofectamine 3000 (Invitrogen, Carlsbad, California, USA). The fluorescence intensity of the cells was detected under a fluorescence microscope at 24 h posttransfection to measure the cell transfection rate.

### 2.4. Recombinant Adeno-Associated Virus 9- (rAAV9-) Mediated Gene Delivery in the Heart

The mice that had operation were randomly selected and divided into 5 groups (*n* = 10) including the sham group, MI group, rAAV9-PCDNA-LINC00261 group (PCDNA-LINC00261), rAAV9-pcDNA-TNRC6A group (pcDNA-TNRC6A), and combined group (expressing PCDNA-LINC00261, miR-522-3p, and pcDNA-TNRC6A). Next, 20 *μ*l rAAV9 vectors containing 5 × 10^12^ genome copies (GC) were injected in five randomly selected sites within left ventricles. Mice in the sham and MI groups were injected with rAAV9 vectors. The expression levels of LINC00261, miR-522-3p, and TNRC6A in the myocardium were measured 1 week after injection.

### 2.5. RNA In Situ Hybridization (ISH) and Fluorescence In Situ Hybridization (FISH)

*In situ* detection of LINC00261 transcription was performed using the RNAscope kit (Advanced Cell Diagnostics, Hayward, CA, USA). A horseradish peroxidase-based signal amplification system was used for hybridization to the target probes, followed by color development with 3,3′-diaminobenzidine. Positive staining was determined by brown punctate dots in the nucleus and/or cytoplasm. RNA-FISH was performed as previously described [23].

### 2.6. RT-qPCR

Total RNAs were extracted by Trizol (Invitrogen, USA). TIANScript II RT Kit (Tiangen, Beijing) was used to reverse transcribe RNAs into cDNAs. RT-qPCR reactions were prepared using the RealMastcrMix (SYBR Green, Tianjian Biotechnology Co., Ltd., Beijing, China). The relative expression levels of LINC00261 and TNRC6A were normalized to the internal reference GAPDH using the 2^-*ΔΔ*Ct^ method, while the expression levels of miR-522-3p were normalized to U6-snRNA. The primer sequences used in this study are listed in Table 1.

### 2.7. Dual-Luciferase Assay

The fragment of 3′-UTR of LINC00261 and TNRC6A was synthesized and fused with pmiRRB-REPORORTTM vector (Libobio, China) to construct LINC00261 and TNRC6A 3′-UTR mutant vectors. H9c2 cells cultured for 24 h were cotransfected with vectors (LINC00261/TNRC6A in the wide type (WT) or mutant (MT)), and luciferase reporter gene plasmid and miR-522-3p mimics were transfected using FugeneHD transfection reagent (Promega, Madison, WI, USA) for 48 h. After washing with PBS and lysing with 80 *μ*l of lysis buffer, cells were collected to detect luciferase strength by firefly luciferase and sea cucumber luciferase substrates separately. Luciferase intensity was measured with a microplate reader (Thermo Fisher Scientific, USA).

### 2.8. MTT Assay

The transfected cardiomyocytes were cultured in 6-well plates for 0, 24, 48, and 72 h and then treated with 20 *μ*l MTT (Servicebio, China) and 5% CO_2_ at 37°C for 4 h. The solution was then aspirated, passed through a shaker with 100 *μ*l dimethyl sulfoxide (Sigma, USA), and mixed gently for 10 min to dissolve crystals. The optical density (OD) values were measured with absorbance at 570 nm using a microplate. Cell survival rate was recorded.

### 2.9. Flow Cytometry

Cardiomyocytes after MI were digested with trypsin at 72 h posttransfection, followed by centrifugation at 1,000 rpm for 5 min. Cells were cleaned twice and centrifuged at 1,000 rpm for 5 min. The collected cells were fixed with 1 ml ethanol at 4°C overnight. Then, 100 *μ*l of RNase A (Solarbio, China) was added to incubate the cells at 37°C for 30 min in the dark. Propidium iodide (50 *μ*l) was then added. After incubation for 1 h in the dark, cells were detected by flow cytometry.

### 2.10. Western Blotting

RIPA (Beyotime, China) were used to extract proteins from H9c2 cells. Protein concentrations were determined using BCA (BioTeke, China). After separation on SDS-PAGE, proteins were transferred to a polyvinylidene fluoride (PDVF) membrane. The membrane was then treated with 5% skimmed milk for 1 h. Then, the membrane was incubated with anti-TNRC6A (1 : 500, Abcam, USA), anticleaved caspase-3 (1 : 500, Abcam, USA), anti-BCL2 (1 : 1,000, Abcam, USA), anti-Bax (1 : 1,000, Abcam, USA), anti-cyclinD1 (1 : 1,000, Abcam, USA), and GAPDH anti-cyclinD1 (1 : 1,000, Abcam, USA) at 4°C overnight, followed by further incubation with goat anti-rabbit IgG H and L-HRP (1 : 5,000, Abcam, USA). SuperECL (Applygen, China) was used to detected membrane-bound immune complexes. GAPDH was used as an internal control.

### 2.11. Statistical Analyses

Statistical analyses were performed using SPSS 17.0 software. Data were expressed as mean ± standard deviation (SD). Comparisons between two groups were performed using independent sample *T*-tests. Three replicates were performed and compared. The one-way ANOVA test followed by the Bonferroni post hoc test was performed to analyze the differences among multiple groups. *P* < 0.05 was considered as statistically significant.

## 3. Results

### 3.1. LINC00261 and TNRC6A Were Upregulated, While miR-522-3p Was Downregulated in Coronary Heart Disease Tissues after MI

**Figure 1:**
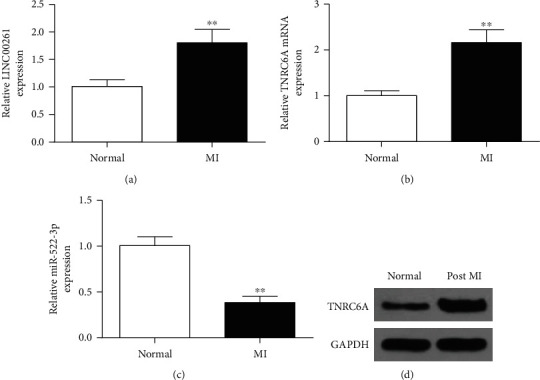
Changes in the expression levels of LINC00261, TNRC6A, and miR-522-3p in cells after MI. (a) Relative expression levels of LINC00261 in normal cells and cells after MI. (b) The expression of TNRC6A in normal cells and cells after MI. (c) Relative expression levels of miR-522-3p in normal cells and cells after MI. Data are expressed as mean ± SD. *N* = 3. ^∗∗^*P* < 0.01, compared to normal cells. (d) Western blot results of expression of TNRC6A in normal cells and cells after MI.

The expression levels of LINC00261, miR-522-3p, and TNRC6A in MI and normal cells were evaluated by RT-qPCR. The results showed that compared to normal cells, the expression levels of LINC00261 significantly increased in myocardial tissues after MI, while the expression levels of miR-522-3p significantly decreased (*P* < 0.01) (Figures 1(a) and 1(c)). In addition, compared with normal cells, TNRC6A was significantly upregulated in cells after MI (*P* < 0.01) (Figure 1(b)). TNRC6A was also induced after MI (Figure 1(d)). These results demonstrated that in cells after MI, LINC00261 and TNRC6A were upregulated, while miR-522-3p was downregulated.

### 3.2. Knockdown of LINC00261 Could Increase the Viability of Cardiomyocytes and Inhibit Cell Apoptosis

**Figure 2:**
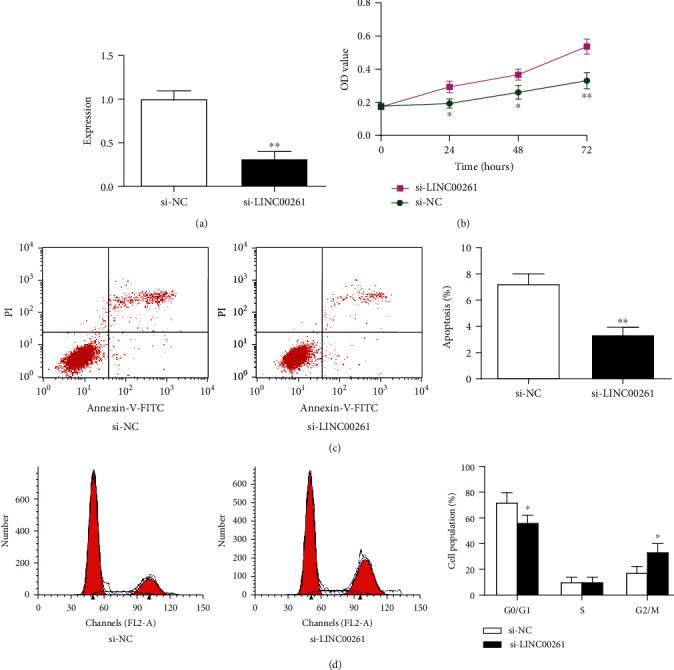
Effect of si-LINC00261 on cardiomyocyte proliferation, apoptosis, and cell cycle. (a) The expression of LINC00261 in cells transfected with si-NC or si-LINC00261. (b) The cell survival rate of the si-LINC00261 group in contrast to si-NC was much better than that of the NC group. (c) Cell apoptosis in cells transfected with si-NC or si-LINC00261. (d) Phase change in transfected cells with si-NC or si-LINC00261. Data are expressed as mean ± SD. *N* = 3. ^∗^*P* < 0.05, ^∗∗^*P* < 0.01, compared with the si-NC group.

RT-qPCR results showed that after transfection with si-LINC00261, the expression of LINC00261 was significantly inhibited (*P* < 0.01) (Figure 2(a)). After transfection with si-LINC00261, the viability of cardiomyocytes was significantly enhanced after 1 day (*P* < 0.01), 2 days, and 3 days (*P* < 0.01) (Figure 2(b)). In addition, flow cytometry results showed that myocardial apoptosis was significantly inhibited after transfection with si-LINC00261 (*P* < 0.01) (Figure 2(c)). Furthermore, the si-LINC00261 group had significantly fewer G0/G1 cardiomyocytes than that in the si-NC group (*P* < 0.05), while the si-LINC00261 group had significantly increased G2/M cardiomyocytes (*P* < 0.01) (Figure 2(d)). These results indicated that knockdown of LINC00261 increased the survival of cardiomyocytes by inhibiting apoptosis and promoting cell cycle phase change.

### 3.3. LINC00261 Targeted miR-522-3p in Cardiomyocytes

**Figure 3:**
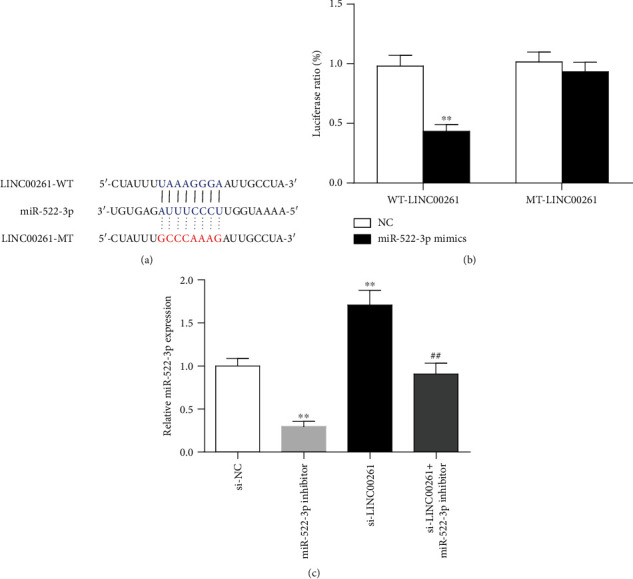
LINC00261 targeted miR-522-3p. (a) Binding sequences between miR-522-3p and LINC00261. (b) Luciferase activity between NC or miR-522-3p mimics and the wild type or mutant LINC00261. ^∗∗^*P* < 0.01, compared to NC. (c) The expression of miR-522-3p in cells transfected with si-NC, miR-522-3p inhibitor, si-LINC00261, or miR-522-3p inhibitor+si-LINC00261. ^∗∗^*P* < 0.01 compared to the si-NC group; data are expressed as mean ± SD. *N* = 3. ^##^*P* < 0.01 compared to the si-LINC00261 group.

The binding sequence between LINC00261 and miR-522-3p is shown in Figure 3(a). Luciferase reporter assay results demonstrated that cotransfection of miR-522-3p mimic and LINC00261 wild type resulted in the lowest luciferase activity (*P* < 0.01) in all treatments (Figure 3(b)). Moreover, RT-qPCR results illustrated that knockdown of LINC00261 resulted in increased expression levels of miR-522-3p in myocardial cells (*P* < 0.01) (Figure 3(c)), indicating that LINC00261 could bind with miR-522-3p in cardiomyocytes after MI.

### 3.4. miR-522-3p Targeted TNRC6A in Cardiomyocytes

**Figure 4:**
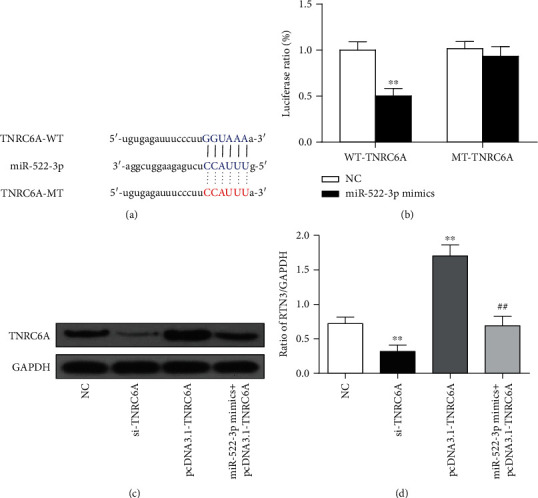
TNRC6A was targeted by miR-522-3p. (a) Binding sequences between miR-522-3p and TNRC6A. (b) Luciferase activity between NC, miR-522-3p mimics, and the wild type or mutant of TNRC6A. (c, d) The expression of TNRC6A protein in cells transfected with NC, si-TNRC6A, pcDNA3.1-TNRC6A, or miR-522-3p mimics+pcDNA3.1-TNRC6A. Data are expressed as mean ± SD. *N* = 3. ^∗∗^*P* < 0.01 compared with the NC group; ^##^*P* < 0.01 compared with the pcDNA3.1-TNRC6A group.

The binding sequences between miR-522-3p and TNRC6A are shown in Figure 4(a). The luciferase reporter assay results demonstrated that compared to the NC group, the ratio of luciferase cotransfected with miR-522-3p mimic and WT TNRC6A was lower (*P* < 0.01), while there was no difference in the TNRC6A mutant (*P* > 0.05) (Figure 4(b)). Luciferase reporter assay results indicated that the binding site of miR-522-3p to TNRC6A was unique. Western blotting analysis was performed to identify the relationships between miR-522-3p and TNRC6A *in vitro*. After cotransfection with miR-522-3p mimic and TNRC6A, the expression levels of TNRC6A were similar to that of NC (*P* < 0.01) (Figures 4(c) and 4(d)). These results indicated that miR-522-3p could inhibit the expression of TNRC6A *in vitro.*

### 3.5. TNRC6A Regulated Cell Viability and Apoptosis of Cardiomyocytes after MI

**Figure 5:**
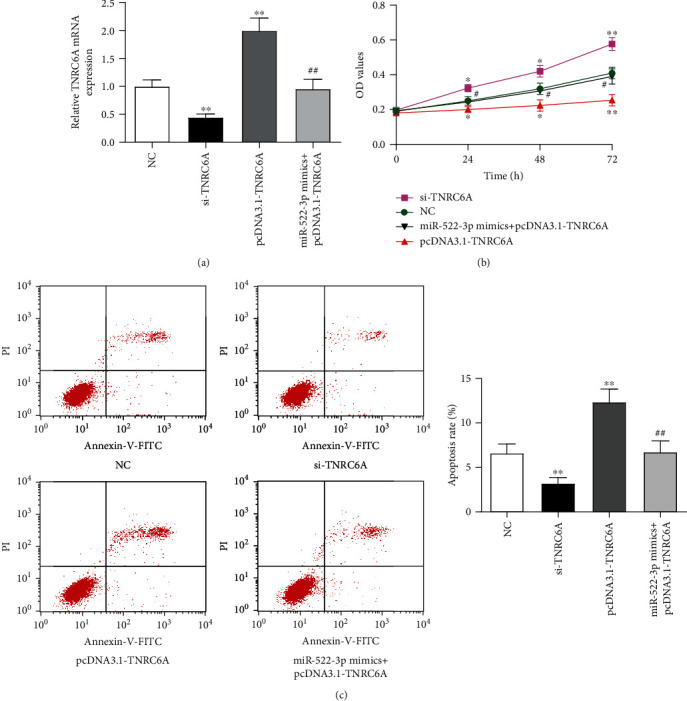
Effect of TNRC6A on myocardial cell proliferation and death. (a) The expression of TNRC6A in cells transfected with NC, si-TNRC6A, pcDNA3.1-TNRC6A, or miR-522-3p mimics+pcDNA3.1-TNRC6A. (b) Cell viability in cells transfected with NC, si-TNRC6A, pcDNA3.1-TNRC6A, or miR-522-3p mimics+pcDNA3.1-TNRC6A. (c) Cell apoptosis rate in cells transfected with NC, si-TNRC6A, pcDNA3.1-TNRC6A, or miR-522-3p mimics+pcDNA3.1-TNRC6A. *N* = 3. ^∗^*P* < 0.05, ^∗∗^*P* < 0.01, compared to the NC group; compared to the pcDNA3.1-TNRC6A group, ^#^*P* < 0.05, ^##^*P* < 0.01.

**Figure 6:**
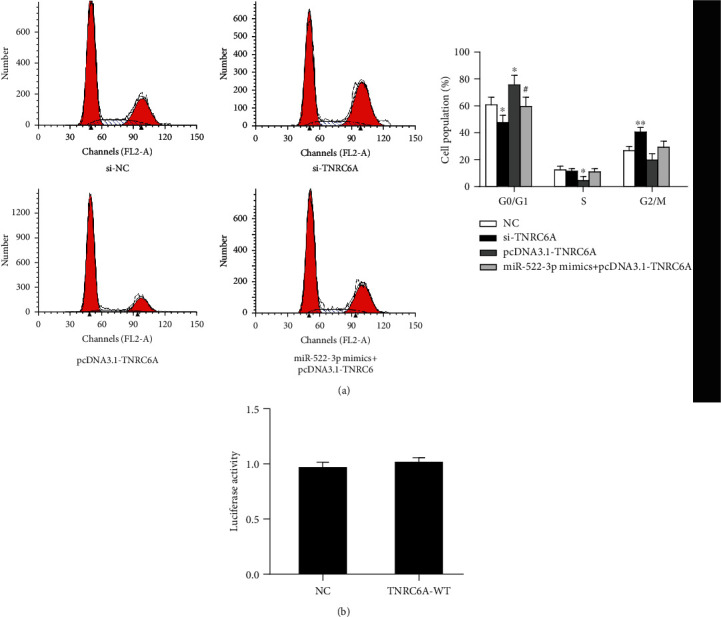
Effect of TNRC6A on myocardial cell cycle. (a) Phase change in cells transfected with NC, si-TNRC6A, pcDNA3.1-TNRC6A, or miR-522-3p mimics+pcDNA3.1-TNRC6A. *N* = 3. ^∗^*P* < 0.05, ^∗∗^*P* < 0.01, compared to the NC group; compared to the pcDNA3.1-TNRC6A group. *N* = 3. ^#^*P* < 0.05. (b) Luciferase assay results for LINC00261-Luc report system transfected by TNRC6A.

RT-qPCR results showed that the expression of TNRC6A was markedly suppressed after si-TNRC6A treatment, and the expression of TNRC6A was induced after the transfection of pcDNA3.1-TNRC6A (*P* < 0.01) (Figure 5(a)). After si-TNRC6A transfection, the viability of cardiomyocytes was significantly enhanced after 1 day (*P* < 0.01), 2 days, and 3 days (*P* < 0.01) (Figure 5(b)). PcDNA3.1-TNRC6A treatment could significantly inhibit cell viability after 1 day (*P* < 0.01), 2 days, and 3 days (*P* < 0.01) (Figure 5(b)). Flow cytometry results showed that compared with the NC group, there was no significant difference in the apoptosis rate of the miR-522-3p mimic+pcDNA3.1-TNRC6A group (*P* > 0.05) (Figure 5(c)). However, among the four groups, the si-TNRC6A group had the lowest apoptotic rate (*P* < 0.05), and the pcDNA3.1-TNRC6A group had the highest apoptotic rate (*P* < 0.01) (Figure 5(c)). In addition, the si-TNRC6A group had significantly fewer G0/G1 cardiomyocytes than the NC group (*P* < 0.05), while in the si-TNRC6A group, G2/M cardiomyocytes had a significant increase (*P* < 0.01) (Figure 5(c)). But pcDNA3.1-TNRC6A can arrest cell phase in the G0/G1 phase (*P* < 0.05) (Figure 5(c)). Compared to the NC group, G0/G1 phase cardiomyocytes in the pcDNA3.1-TNRC6A group were blocked (Figure 6(a)). To examine whether the reverse effect was due to the direct binding between TNRC6A and LINC00261, a luciferase reporter assay was conducted to detect the possible binding between TNRC6A and LINC00261; the results showed no binding potential between them (Figure 6(b)). In summary, miR-522-3p mimics effectively reversed the viability or apoptosis of myocardial cells induced by pcDNA3.1-TNRC6A after MI, and the reversed effect was independent of LINC00261.

### 3.6. LINC00261 Could Regulate the Viability of Cardiomyocytes and Cell Apoptosis *In Vivo*

**Figure 7:**
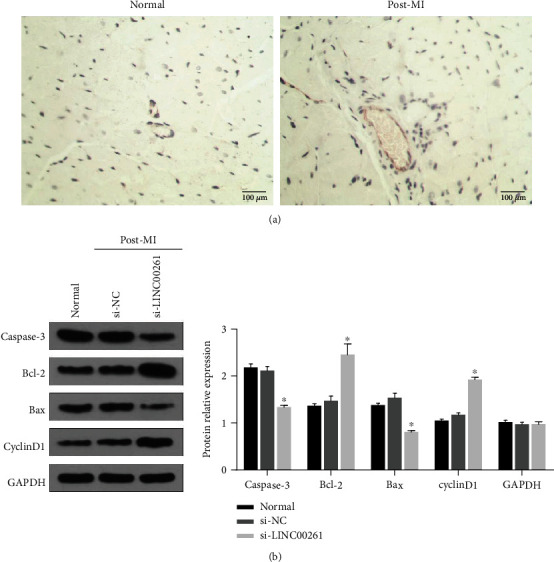
LINC00261 could regulate the viability of cardiomyocytes and cell apoptosis *in vivo*. (a) RNA-FISH results of the expression of LINC00261 in the heart from normal and MI mouse, respectively. Scale bar = 100 *μ*m. (b) Western blotting results of the expression of Active CASPASE3, BCL-2, BAX, and CYCLIND1 from the normal group, the post-MI+AAV-siNC group, and the post-MI+AAV-si LINC00261 group mice, respectively. *N* = 3. ^∗^*P* < 0.05, compared to the post-MI+AAV-siNC group.

To identify the function of LINC00261 *in vivo*, RNA scope was used to detect the expression of LINC00261 post-MI *in situ*, and the results showed that LINC00261 was mainly expressed in myocardial cells but not the epithelial cells (Figure 7(a)). Furthermore, AAV-si LINC00261 was also delivered into MI mice. Consistent with the *in vitro* results, Western blotting results of apoptosis-related markers indicated that si-LINC00261 attenuated cell apoptosis (Figure 7(b)). AAV-PCDNA-LINC00261 was also delivered into MI mice, the cell apoptosis was improved, and cell cycle G0/G1 for cardiomyocytes was also arrested (Figure S1).

### 3.7. The LINC00261-miR-522-3p/TNRC6A Axis Regulated Apoptosis of Myocardial Cells in the MI Model

**Figure 8:**
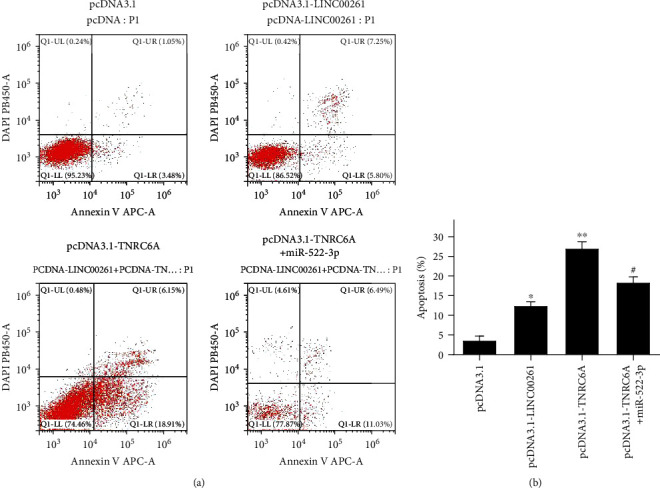
The LINC00261-miR-522-3p/TNRC6A axis regulated apoptosis of myocardial cells in the MI model. (a) Apoptosis rate for myocardial cells which were transfected with AAV-PCDNA, AAV-PCDNA-LINC00261, AAV-PCDNA-LINC00261+TNRC6A, or AAV-PCDNA-LINC00261+miR-522-3p mimics+pcDNA3.1-TNRC6A, respectively. (b) Statistic of the apoptosis ratio. Each experiment was repeated 3 times independently. *N* = 3. ^∗^*P* < 0.05, ^∗∗^*P* < 0.01, compared to the PCDNA group; ^#^*P* < 0.05, compared to AAV-PCDNA-LINC00261+TNRC6A.

To identify the role of the LINC00261-miR-522-3p/TNRC6A axis in the process of MI protection, series of hearts from the pcDNA-LINC00261 overexpression group, PCDNA-LINC00261+PCDNA-TNRC6A group, and PCDNA-LINC00261+PCDNA-TNRC6A+miR-522-3p mimic group post-MI were prepared. Furthermore, a flow cytometry assay was performed to detect the apoptosis ratio of cells in the above groups (Figure 8). These results indicated that cell apoptosis was improved by overexpression of LINC00261, augmented by TNRC6A but rescued by miR-522-3p.

## 4. Discussion

Previous studies have established the role of various lncRNAs such as lncRNA MIAT [24], lncRNA KCNQ1OT1 [25], and lncRNA CAIF [26] in MI. These studies demonstrate that lncRNAs are widely involved in signal transmission and affect cell metabolism, growth, differentiation, apoptosis, and death [27, 28]. They also play important roles in the occurrence and development of cardiovascular disease [29, 30]. Our study investigated the expression levels of LINC00261 in MI samples. We found that the expression levels of LINC00261 were significantly increased in myocardial tissues after MI. In addition, the viability of cardiomyocytes was significantly enhanced after transfection with si-LINC00261. Myocardial apoptotic cells were significantly inhibited after transfection with si-LINC00261. For the first time, we here reported that the expression of LINC00261 was upregulated in cardiomyocytes after MI, and knockdown of LINC00261 can increase the viability of cardiomyocytes and inhibit cell apoptosis.

miR-522-3p was demonstrated to play promotive roles or tumor-suppressive roles in different types of human cancer, such as colorectal cancer [31] and lung cancer [19]. However, for cardio diseases, very limited information has been revealed. In our study, we found that the expression levels of miR-522-3p were significantly decreased in MI cells and tissues compared with that in normal cells and tissues. A recent study reported the targeting effect between LINC00261 and miR-522-3p [19]. In our study, both bioinformatics analyses and luciferase reporter assay showed that LINC00261 could target miR-522-3p. RT-qPCR results showed that knockdown of LINC00261 resulted in an increased expression level of miR-522-3p in myocardial cells. Consistent with previous study, we found that LINC00261 targets miR-522-3p in the myocardium after MI.

The C-terminal domain of human TNRC6A is an Argonaute-navigator protein for miRNA-mediated gene silencing in the nucleus [20]. A recent study reported that the expression of TNRC6A is dysregulated during MI [21]. In addition, several miRNAs could bind with TNRC6A to exert the combinational effect on different cells [20, 21]. In our study, we found that TNRC6A was significantly upregulated in cells after MI compared with that in normal cells. Moreover, luciferase activity and Western blotting results confirmed that miR-522-3p could bind with TNRC6A in cardiomyocytes. To the best of our knowledge, we are the first to report that miR-522-3p targets TNRC6A in cardiomyocytes and the binding of miR-522-3p and TNRC6A results in downregulation of TNRC6A in cardiomyocytes.

TNRC6 was shown as a master regulator of miRNA functions in MI [32]. Silencing of TNRC6a led to hypoxia-induced upregulation of miR-15b, miR-21, miR-26, miR-34a, and miR-140, not mirrored by downregulation of their target proteins [32]. In our study, we found that the viability of cardiomyocytes was significantly enhanced after si-TNRC6A transfection. The treatment of TNRC6A could significantly inhibit cell viability. Moreover, the si-TNRC6A group had significantly fewer G0/G1 cardiomyocytes, while the si-TNRC6A group had a significant increase in G2/M cardiomyocytes than the NC group. TNRC6A regulates cell viability and apoptosis of cardiomyocytes after MI, and miR-522-3p effectively reversed the viability or apoptosis of myocardial cells after MI induced by TNRC6A.

## 5. Conclusions

LINC00261 downregulated miR-522-3p, and TNRC6A was a direct target of miR-522-3p. Our results indicated that LINC00261 could serve as a therapeutic agent for the treatment of MI.

## Figures and Tables

**Table 1:** Sequences of primers utilized in qRT-PCR.

Name	Forward primer (5′-3′)	Reversed primer (5′-3′)
LINC00261	GTCAGAAGGAAAGGCCGTGA	TGAGCCGAGATGAACAGGTG
TNRC6A	TCACATCATATCACATTGCCAGG	TATGGTTGTCTTGCTCTCTGTCTC
GAPDH	TATGATGATATCAAGAGGGTAGT	TGTATCCAAACTCATTGTCATAC
miR-522-3p	GGGCTCTAGAGGGAAGCGC	CAGTGCGTGTCGTGGAGT
U6	CTTCGGCAGCACATATACT	AAAATATGGAACGCTTCACG

## Data Availability

The datasets generated and/or analyzed during the current study are not publicly available due to research design but are available from the corresponding author on reasonable request.

## Abbreviations

FISH: Fluorescence in situ hybridization
GC: Genome copies
ISH: In situ hybridization
lncRNA: Long noncoding RNA
miRNAs: MicroRNAs
MI: Myocardial infarction
NC: Negative control
TNRC6A: Trinucleotide repeat-containing gene 6a
3′-UTR: 3′-Untranslated region.

## Supplementary Materials

Supplementary Materials Supplement Figure 1: effect of LINC00261 on cardiomyocyte apoptosis and cell cycle after MI. (A) Cell apoptosis was detected in pcDNA and PCDNA-LINC00261 transfected H9C2 cells after MI. (B) Phase change is transfected with pcDNA or PCDNA LINC00261 after MI. Compared with the PCDNA group, ^∗∗^*P* < 0.01.
