# Supplementary material for: Downregulation of Long Noncoding RNA LINC00261 Attenuates Myocardial Infarction through the miR-522-3p/Trinucleotide Repeat-Containing Gene 6a (TNRC6A) Axis
Source: Cardiovasc Ther. 2021 Jun 18;2021:6628194. doi: 10.1155/2021/6628194 (PMC8235986; doi:10.1155/2021/6628194)
Supplement: Supplementary Materials — Supplement Figure 1: effect of LINC00261 on cardiomyocyte apoptosis and cell cycle after MI. (A) Cell apoptosis was detected in pcDNA and PCDNA-LINC00261 transfected H9C2 cells after MI. (B) Phase change is transfected with pcDNA or PCDNA LINC00261 after MI. Compared with the PCDNA group, ∗∗P < 0.01. [file 6628194.f1.docx]

**Figure S1**

**
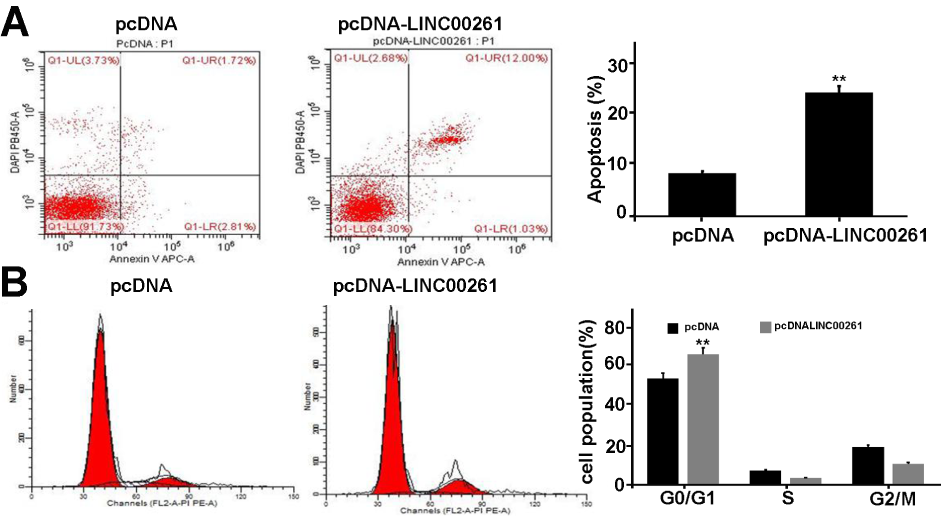
**

**Supplement Figure 1. Effect of LINC00261 on cardiomyocyte apoptosis and cell cycle after MI.(A)** cell apoptosis was detected in pcDNA and PCDNA-LINC00261 transfected H9C2 cells after MI **(B)** Phase change in transfected withpcDNA or PCDNA LINC00261 after MI. Compared with PCDNA group,, ** P <0.01
